# Instability of non-REM sleep in older women evaluated by sleep-stage transition and envelope analyses

**DOI:** 10.3389/fnagi.2022.1050648

**Published:** 2022-12-06

**Authors:** Insung Park, Chihiro Kokudo, Jaehoon Seol, Asuka Ishihara, Simeng Zhang, Akiko Uchizawa, Haruka Osumi, Ryusuke Miyamoto, Kazumasa Horie, Chihiro Suzuki, Yoko Suzuki, Tomohiro Okura, Javier Diaz, Kaspar E. Vogt, Kumpei Tokuyama

**Affiliations:** ^1^International Institute for Integrative Sleep Medicine (WPI-IIIS), University of Tsukuba, Tsukuba, Japan; ^2^Graduate School of Comprehensive Human Science, University of Tsukuba, Tsukuba, Japan; ^3^Faculty of Health and Sports Sciences, University of Tsukuba, Tsukuba, Japan; ^4^Japan Society for the Promotion of Science, Tokyo, Japan; ^5^Center for Computational Sciences, University of Tsukuba, Tsukuba, Japan; ^6^R&D Center for Tailor-Made QOL, University of Tsukuba, Tsukuba, Japan

**Keywords:** delta power, sleep transition, envelope analysis, K-complexes, SWS, older

## Abstract

**Study objective:**

Traditionally, age-related deterioration of sleep architecture in older individuals has been evaluated by visual scoring of polysomnographic (PSG) recordings with regard to total sleep time and latencies. In the present study, we additionally compared the non-REM sleep (NREM) stage and delta, theta, alpha, and sigma wave stability between young and older subjects to extract features that may explain age-related changes in sleep.

**Methods:**

Polysomnographic recordings were performed in 11 healthy older (72.6 ± 2.4 years) and 9 healthy young (23.3 ± 1.1 years) females. In addition to total sleep time, the sleep stage, delta power amplitude, and delta, theta, alpha, and sigma wave stability were evaluated by sleep stage transition analysis and a novel computational method based on a coefficient of variation of the envelope (CVE) analysis, respectively.

**Results:**

In older subjects, total sleep time and slow-wave sleep (SWS) time were shorter whereas wake after sleep onset was longer. The number of SWS episodes was similar between age groups, however, sleep stage transition analysis revealed that SWS was less stable in older individuals. NREM sleep stages in descending order of delta power were: SWS, N2, and N1, and delta power during NREM sleep in older subjects was lower than in young subjects. The CVE of the delta-band is an index of delta wave stability and showed significant differences between age groups. When separately analyzed for each NREM stage, different CVE clusters in NREM were clearly observed between young and older subjects. A lower delta CVE and amplitude were also observed in older subjects compared with young subjects in N2 and SWS. Additionally, lower CVE values in the theta, alpha and sigma bands were also characteristic of older participants.

**Conclusion:**

The present study shows a decrease of SWS stability in older subjects together with a decrease in delta wave amplitude. Interestingly, the decrease in SWS stability coincided with an increase in short-term delta, theta, sigma, and alpha power stability revealed by lower CVE. Loss of electroencephalograms (EEG) variability might be a useful marker of brain age.

## Introduction

Epidemiologic studies indicate that sleep disorders are associated with chronic diseases such as obesity, diabetes heart disease as well as poor mental health (Foley et al., 2004, 1995). Older adults voice more complaints about sleep than young adults (Wolkove et al., 2007; Ministry of Health, Labor and Welfare, 2019), and over 50% of older adults express chronic sleep complaints (Ancoli-Israel, 2009). With advancing age, the characteristic sleep experience changes are as follows: (1) advanced sleep phase (e.g., go to bed early and rise early), (2) longer sleep onset latency, (3) shorter overall sleep duration, (4) increased sleep fragmentation, (5) reduced amount of slow-wave sleep (SWS), and (6) increased number of wake events and time spent awake (Prinz et al., 1990; Ohayon et al., 2004).

Age-related changes in sleep can be viewed as the effect of aging on homeostatic and circadian sleep processes (Borbély, 1982; Borbély et al., 2016). The magnitude of the delta power in the electroencephalograms (EEG) depends on the awake time before sleep onset, and is considered an index of a homeostatically regulated process, i.e., Process S of the two-process model of sleep (Borbély, 1982). Previous studies targeting various age groups showed an attenuation of delta power with aging (Carrier et al., 2001; Gaudreau et al., 2001; Landolt and Borbély, 2001; Darchia et al., 2007). The effect of aging on the circadian process of sleep is reflected as an advanced sleep phase. Indeed, the circadian rhythm of body temperature also shows a phase advance as well as a decreased amplitude (Weitzman et al., 1982; Van Someren et al., 2002). Although sleep is conventionally classified into discrete stages every 30 s, the aggregated deterioration in the sleep architecture of older subjects is most commonly assessed as total time in each sleep stage and sleep onset latency; few studies have evaluated differences in the stability of sleep stages and their transitions. Given the homeostatic and circadian components of sleep regulation, it is important to evaluate the time course of the sleep stages and delta power throughout the entire sleeping period.

Hypnograms showing sleep stages as a function of time could provide insight into sleep quality, such as the number and duration of different sleep stages, and sleep stage transitions (Laffan et al., 2010). For example, it is well known that exercise performed during the day increases the probability of transitions from N1 to N2 and decreases transitions from N1 to waking (Kishi et al., 2013). Comparisons of hypnograms between young and older subjects are limited, however, and the sleep stage durations have not been considered (Schlemmer et al., 2015).

A novel computational analysis method of EEG, termed the envelope analysis, was proposed by Díaz et al. (2018), as a complement to classic spectral analysis. The EEG is band-pass filtered for the desired frequency range—for example, the delta band (0.5–4 Hz), and the envelope of this signal is obtained. The amplitude of the envelope can be thought of as the instantaneous energy in the respective band with its time course revealing the temporal stability of the oscillation in this band. The coefficient of variation of the envelope (CVE) of the given EEG band provides a scale-independent measure of its temporal stability. In human sleep, low delta-CVE values were associated with the stable oscillations characterizing SWS, while high delta-CVE values were a sign of the irregular phasic processes associated with shallow non-rapid eye movement (NREM) sleep, stages N1 and N2 (Díaz et al., 2018; Park et al., 2021).

In the present study, polysomnographic (PSG) recordings were performed in young and older subjects. First, we confirmed previously reported age-related changes in sleep architecture, such as longer wakefulness and shorter SWS, in older subjects. Second, we assessed the stability of NREM sleep, particularly SWS, by comparing the number of episodes and their duration, and by analyzing sleep stage transitions; detecting more transitions in older subjects. Third, after Fourier transform of EEG signals, delta power was compared between age groups, with older subjects exhibiting lower EEG delta power values. Lastly, CVE analysis of the delta, theta, alpha and sigma bands showed significant differences between young and older subjects. Older participants showed lower CVE values, which indicate less short term variability of EEG power in the respective bands.

## Materials and methods

### Participants

All participants were recruited through advertisements, and 11 older women (68–76, average 72.1 years old) and 9 young women (21–25, average 22.3 years old) participated in the study. Inclusion criteria were standard body size (BMI < 30 kg/m^2^), and absence of the following: subjective sleep complaints, use of sleeping pills, exercising habit more than twice a week, smoking habit, and shift work or trans meridian travel within 1 month before the study. In addition, young women with a regular menstrual cycle were selected. Before beginning the study, the nature, purpose, and risks of the study were explained to all subjects and informed written consent was obtained. All study protocols were approved by the local Ethics Committee of the University of Tsukuba (Ref No., Tai 29-29 and Ref No., 30-134), and conducted in accordance with the Helsinki Declaration.

### Protocol

To accustom the subjects to the experimental environment, the experiment was preceded by an adaptation night in the laboratory within 7 days before the experiment, during which electrodes of the PSG recording system were attached to the subjects. For 1 week before the experiment, subjects were instructed to follow their own regular sleep-wake schedule and this was confirmed by a wrist-worn actigraph (GT3X-BT, AMI, VA, USA).

On the experiment day, subjects ate dinner 5 h before bedtime, and then reported to the laboratory. After attaching the PSG electrodes, subjects entered the environment-controlled room where the temperature and humidity of incoming fresh air were maintained at 25.0 ± 0.5°C and 55.0 ± 3.0%, respectively (Fuji Medical Science, Chiba, Japan). Subjects were instructed to maintain a sedentary posture and stay awake till bedtime, which was individually determined based on their habitual bedtime, and sleep time was set 8 h. As for young subjects, their experimental day was set during the follicular phase to eliminate the effects of menstrual cycles on sleep architecture.

### Polysomnography

Sleep was recorded polysomnographically (PSG-1100, Nihon Kohden, Tokyo, Japan). EEG electrodes were placed at six sites (F3/M2, F4/M1, C3/M2, C4/M1, O1/M2, and O2/M1), and two electro-oculograms and one submental electromyogram were adopted and recorded during an 8-h sleep period. The recordings were scored every 30 s for classification into the five sleep stages: wakefulness (W), rapid eye movement (R), NREM sleep stage 1 (N1), NREM sleep stage 2 (N2), and SWS according to the standard criteria (Silber et al., 2007). Sleep efficiency is calculated as sum of Stage N1, Stage N2, Stage N3, and REM sleep, divided by the total time in bed and multiplied by 100. K-complexes are well delineated, sharp negative waves followed by a positive component standing out from the background EEG with a total duration ≥0.5 s. They are usually maximal in amplitude when recorded using frontal derivations (American Academy of Sleep Medicine [AASM], 2010). We identified K complexes through a machine-learning algorithm using the EEG directly as an input. The number of K-complex was normalized as % of 5 s segment in the entire N2, in which K-complexes was detected. The number of sleep episodes, defined as the time interval of consecutive sleep stages, and the length of each episode were calculated for each subject. The C3-A2 EEG recording was analyzed using discrete fast Fourier transformation. The fast Fourier transformation was conducted on an EEG record length of 5 s to obtain a frequency resolution of 0.2 Hz (Park et al., 2017). Each 5-s segment of the EEG signal was first windowed with a Hanning tapering window before computing the power spectrum. The power content of the delta band for each 30-s epoch of sleep was determined as the average delta power across the 6 consecutive 5-s segments of the EEG (expressed as μV^2^).

The CVE for the different EEG bands was calculated for the C3-A2 EEG recordings at 30-s intervals. To minimize aliasing effects, the epochs had a 50% overlap, which means the epoch length was 60 s. First, every epoch was digitally bandpass-filtered (delta: 0.5–4.0 Hz, theta: 4–8 Hz, alpha: 8–12 Hz, sigma 13–17 Hz) with a fourth-order IIR implementation of a Butterworth filter using the “signal” package for the R language^1^ as filt_EEG. The envelope of the filt_EEG (Filt_EEG_env) was obtained using its Hilbert transform (Ht) according to the standard relation:


$$\mathrm{Filt}\,\_\,\mathrm{EEG}\,\_\,\mathrm{env}=\sqrt{\left({\mathrm{Filt}}_{\_}\,{\mathrm{EEG}}^{2}+\mathrm{Ht}\,{\left(\mathrm{filt}\,\_\,\mathrm{EEG}\right)}^{2}\right)}$$

Both the filter and envelope calculations usually produce artifacts at the border of each epoch. To avoid this problem, the samples of each epoch were collected with a 10% excess (i.e., 66 s in total, 3 s per side). Once the envelope was obtained, the time excess was removed. The mean and standard deviation (SD) of the envelope were calculated and a normalized version of the CVE was obtained: SD/(mean × 0.523); with 0.523 being the value for the CVE of Gaussian waves (Díaz et al., 2018). As a consequence, CVE values larger than 1 indicate processes more phasic than Gaussian waves, while CVE values below 1 indicate more sinusoidal processes. For each epoch, the coefficient of variation of the corresponding envelope was stored as a relevant feature (Díaz et al., 2018). As CVE is a scale independent metric, it is useful to complement the information provided by delta CVE with delta amplitude. The density maps shown in Figure 5 were calculated following the algorithms described in Díaz et al. (2018).

### Statistics

To compare the time course of delta power changes, a two-way ANOVA with repeated measures and Bonferroni’s correction for *post-hoc* pair-wise tests were used. An independent *t*-test was used to compare average PSG parameters. A Mann–Whitney *U*-test was used to compare the average sleep episode duration distribution. Data in the figures are presented as means ± SE or means ± SD, which is denoted in the figure legend. All statistical analyses were performed using IBM SPSS statistical software Version 27.0 (IBM Japan, Ltd., Tokyo, Japan). Statistical significance was set at 5% (two-tailed).

## Results

### Subjects

Table 1 shows the participants’ characteristics. Although the older and young groups exhibited a statistically significant height difference, their heights and weights were comparable to mean values by age, as shown in “Statistics Japan.”^2^ The mean habitual bedtime of the older group was approximately 2 h earlier than that of the young group and their wake time was 2.5 h earlier (*p* < 0.001).

**TABLE 1:** Characteristics of the study participants.

		Young	Older	*P*-value
Age	Year	23.3 ± 1.1	72.6 ± 2.4	< 0.001*
Height	cm	161.8 ± 5.5	152.9 ± 5.0	0.001*
Weight	kg	53.9 ± 9.3	51.5 ± 6.0	0.504
BMI	kg/m^2^	20.5 ± 2.6	22.1 ± 3.0	0.230
PSQI		5.7 ± 2.3	6.1 ± 2.3	0.686
Habitual bedtime	hh: mm ± min	0: 04 ± 65	22: 07 ± 40	< 0.001*
Habitual waketime	hh: mm ± min	8: 38 ± 52	5: 56 ± 23	< 0.001*

Values are mean ± SD. Statistically significant differences are indicated by*. SD, standard deviation; PSQI, Pittsburgh sleep quality index.

### Conventional sleep parameters

**FIGURE 1:**
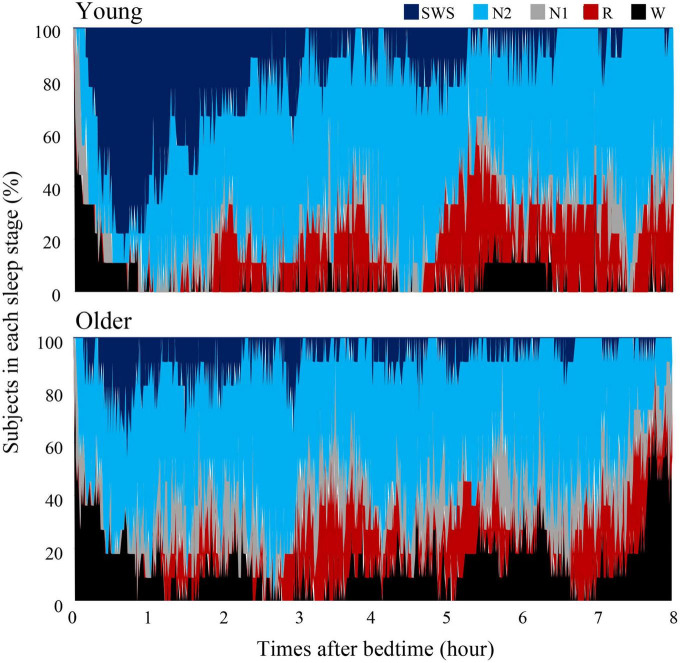
Cumulative display of sleep architecture. Distribution of sleep stages of 9 subjects in the young group and 11 subjects in the older group. The percentage of subjects in each sleep stage is shown; Wake (black), REM sleep (red), NREM Stage 1 (gray), NREM Stage 2 (light blue), and NREM Stage SWS (blue). Data of young group was presented in our previous study to compare sleep architecture of follicular and luteal phase (Zhang et al., 2020).

Overall sleep architecture is summarized in Table 2. Compared with the young subjects, total sleep time and SWS were significantly shorter and wake after sleep onset and N1 were longer in the older subjects. Accordingly, the sleep efficiency was significantly lower in the older group than in the young group. Changes in dominant sleep stage were observed in both age groups; SWS was observed during the first 2 h, and was gradually replaced with N2 and REM in both age groups (Figure 1). The occurrence of K-complexes was calculated in N2 as expressed by the proportions (young subjects: 30.5 ± 6.9% vs. older subjects: 11.2 ± 5.8%, *p* < 0.0001).

**TABLE 2:** Sleep parameters.

		Young	Older	*P*-value
Total bedtime	min	480	480	−
Total sleep time	min	451 ± 27	396 ± 58	0.019*
Wakefulness	min	19 ± 20	72 ± 42	0.003*
Sleep latency	min	11 ± 14	13 ± 19	0.817
Sleep efficiency	%	94 ± 6	82 ± 12	0.019*
N1	min	42 ± 28	73 ± 19	0.008*
N2	min	240 ± 42	224 ± 62	0.507
SWS	min	92 ± 27	36 ± 27	< 0.001*
REM sleep	min	77 ± 15	63 ± 29	0.193
REM sleep latency	min	108 ± 36	122 ± 58	0.527
SWS latency	min	26 ± 17	37 ± 25	0.287
N1 ratio in NREM	%	11 ± 8	23 ± 10	0.009*
N2 ratio in NREM	%	64 ± 7	66 ± 12	0.650
SWS ratio in NREM	%	25 ± 8	11 ± 8	0.001*

Values are mean ± SD. Statistically significant differences are indicated by*.

### Duration and number of sleep episodes in non-REM sleep stages

**FIGURE 2:**
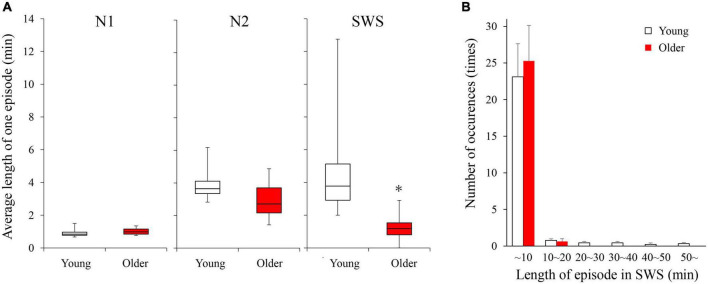
Average length of 1 continuous episode of each NREM stage **(A)** and distribution of SWS sleep episode duration **(B)**. **(A)** A statistically significant difference between older and young groups was detected in the SWS stage with the Mann–Whitney *U*-test (*p* < 0.001). **(B)** Although total numbers of SWS episodes were identical between the 2 age groups, older subjects did not have any episodes longer than 16 min. Asterisk represents a statistically significant difference between the older and young subjects (*p* < 0.05).

Duration and number of NREM sleep episodes were calculated, and statistically significant differences in episode duration were detected between the two age groups in the SWS stage (*p* = 0.230, 0.080, <0.001 for N1, N2, and SWS, respectively) (Figure 2A). Although the duration of most of the SWS episodes was shorter than 10 min in both groups. It was noticeable that younger subjects had episodes longer than that (up to 50 min), while older participants did not have any episodes longer than 16 min (Figure 2B).

### Sleep stage transition

**FIGURE 3:**
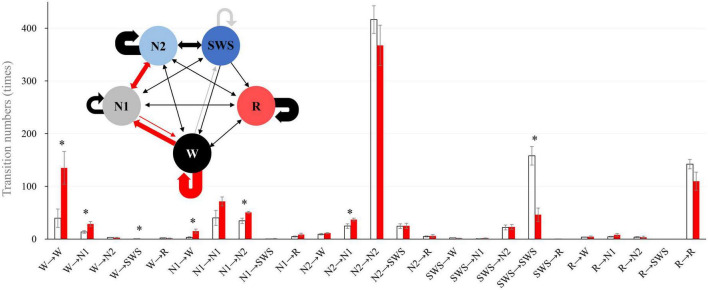
Intra-stage transition numbers and transition probability. Stage transition numbers and diagram during the 8-h experiment night. The young group is shown in white, and the older group in red in the bar graph. Asterisks represent statistically significant differences between older and young groups (*p* < 0.05). In the diagram, the thickness of the arrows indicates the number of transitions (bold line: >100, semi-bold line: >20, thin line ≤20; red and gray arrows indicate statistical significance (red: older >young, and gray: young >older). The I-bar shows SE.

We analyzed sleep stage transitions between all neighboring 30-s epochs. Transition numbers between stages are shown in Figure 3. Transitions from wake to wake, wake to N1, wake to SWS, N1 to wake, N1 to N2, and N2 to N1 were more frequent in older subjects. On the other hand, staying in stage SWS, i.e., SWS to SWS, was significantly less frequently found in older subjects (Figure 3).

### Delta power

**FIGURE 4:**
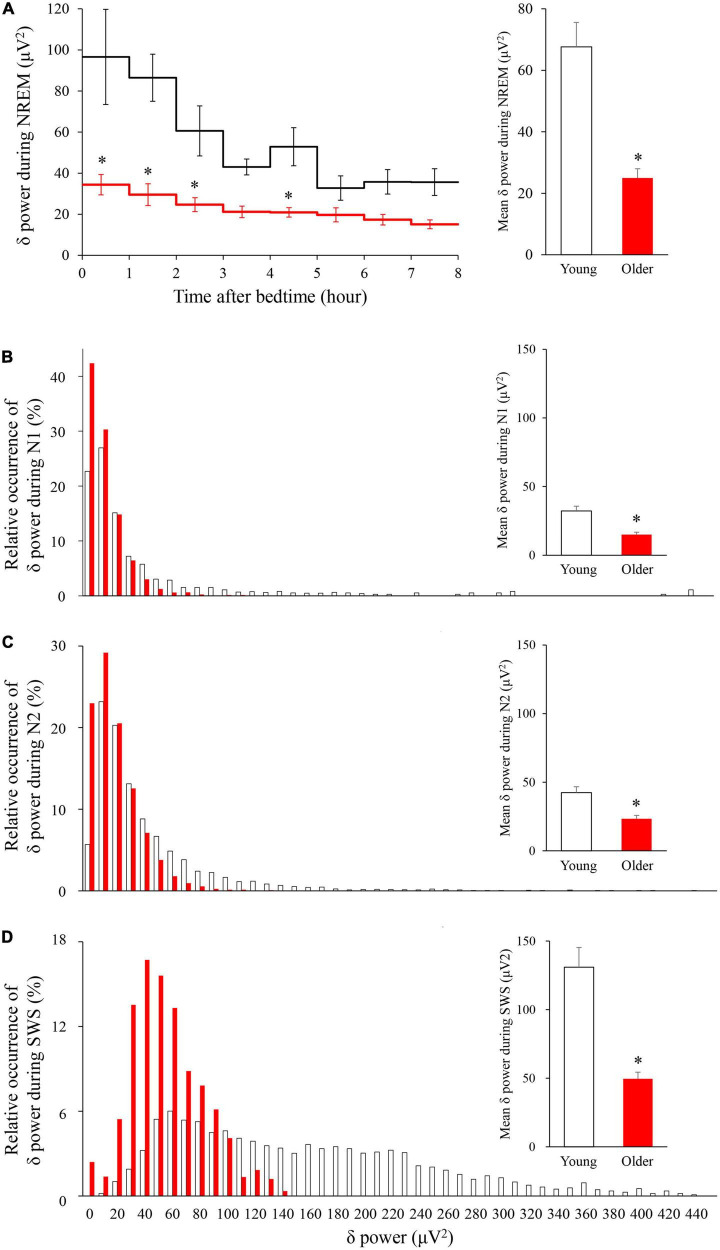
Time course of delta power of NREM sleep EEG **(A)** and relative occurrence of delta power in each NREM stage **(B–D)**. **(A)** An hourly mean ± SE of delta power of the two groups is shown as a line graph and mean delta power during NREM is shown as a bar graph. Hourly mean ± SE is shown for young women (white) and older women (red). The main effect of age, main effect of time, and interaction *p*-values of two-way repeated measures ANOVA were all *p* < 0.001. Asterisks represent statistically significant difference between the two age groups by *post-hoc* pair-wise comparisons using the Bonferroni’s correction (*p* < 0.05). Relative occurrence distribution of 5-s delta power and mean delta power during N1 **(B)**, N2 **(C)**, and SWS **(D)**. Asterisk represents a statistically significant difference between the older and young subjects by an independent *t*-test (*p* < 0.05), and error bars denote the standard error.

The time course of EEG delta power showed significant effects of age group, time, and interaction. Delta power gradually decreased as sleep time increased in both age groups, and delta power was significantly lower in the older group than in the young group (*p* < 0.001) (Figure 4A). The distribution of the delta power was skewed toward lower values for both groups in all NREM stages. Of note, the tendency was even more pronounced for older subjects, especially in SWS. Delta power in each NREM sleep stage and the distribution of delta power for all epochs (30 s) of NREM sleep were also compared. In all NREM stages, mean delta power was significantly lower in the older group than in the younger group (*p* < 0.001, 0.002, and < 0.001 for N1, N2, and SWS, respectively) (Figures 4B–D).

### Delta amplitude vs. delta coefficient of variation of the envelope density maps

**FIGURE 5:**
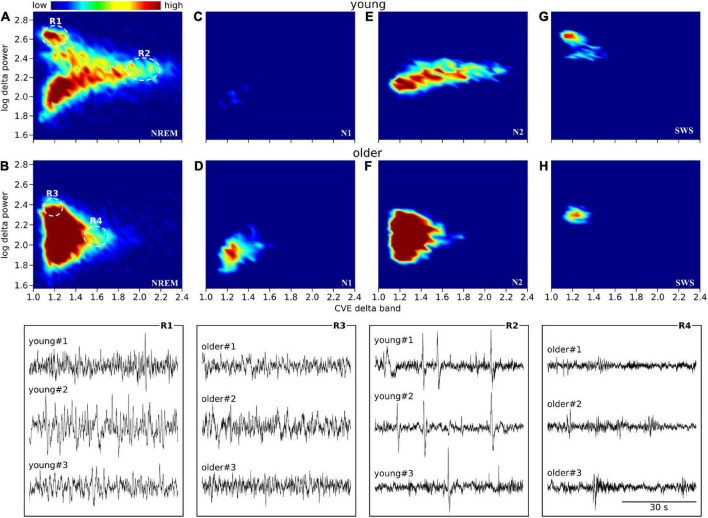
Envelope characterization space (ECS) of EEG delta band during NREM sleep. Upper panels **(A–H)** show the ECS -i.e., CVE vs. amplitude- of the EEG delta band as density plots (2D histograms) of pooled young subjects (first row) and older subjects (second row). Panels **(A,B)** correspond to the ECS considering all NREM epochs. Panels **(C,D)** only N1 epochs. Panels **(E,F)** only N2 epochs. Panels **(G,H)** only SWS (N3) epochs. Notice that while in older subjects all epochs are condensed in a single cluster **(B)**, younger subjects generate rich clustering patterns with evident local densities and a long tail pointing to high CVE values. Bottom traces correspond to representative EEG epochs from three young and three older subjects taken from regions 1 to 4 as indicated in panels **(A,B)**. In young subjects, SWS is an objectively isolated cluster of high amplitude delta waves (R1 vs. R3). In young subjects, high amplitude transients (K complexes) are observed while in the older subjects, the expected amplitude of transient delta activity is greatly diminished (R2 vs. R4).

Scatter plots between the delta power amplitude and delta power CVE are shown as a heatmap-like presentation (Figure 5). Different clusters in NREM were clearly observed between the young and older subjects (Figures 5A,B). When separately analyzed in each NREM stage, a lower CVE and amplitude were observed in older subjects compared with young subjects in N2 and SWS (Figures 5E–H). The ascending order of amplitudes of delta waves was N1, N2, and SWS in both age groups. The delta wave amplitudes of N2 and SWS were clearly separated in young subjects, whereas there was an overlap in older subjects. The lower SWS delta power amplitudes in older subjects explain the difference in the cluster of NREM epochs with a higher amplitude (> 2.4) and lower CVE (< 1.2) between age groups. The cluster along the *X*-axis in N2 was more spread out in young subjects compared with older subjects; this is related to the presence of prominent non-Gaussian (i.e., phasic) delta activity in younger subjects, which is absent in older participants.

### Coefficient of variation of the envelope in the other bands

**FIGURE 6:**
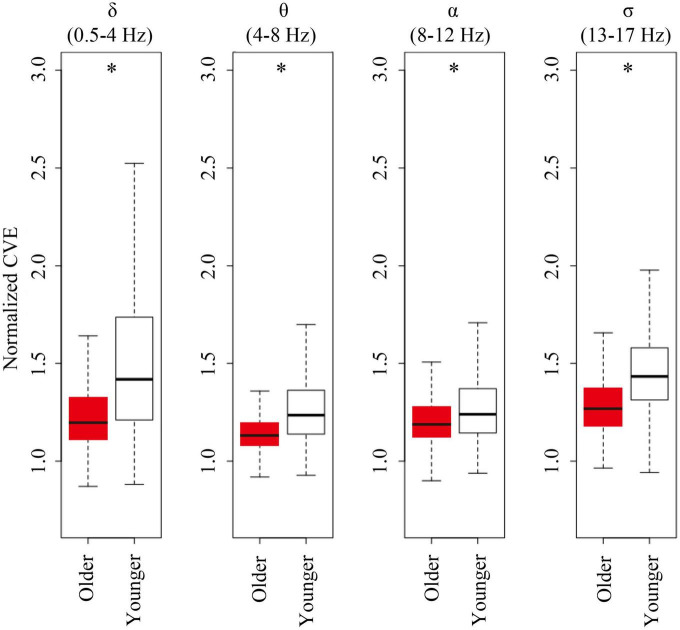
Envelope analysis of all major spectral bands. Box and whisker plots for each band of envelope analysis. The older group is shown in red, and young group is shown in white in the box. A black line in the box to indicate the mean value, a box to indicate variability (± SE), and whiskers around the box to indicate the 95% normal confidence interval for mean. Asterisks represent statistically significant differences between older and young groups (*p* < 0.05).

For all analyzed bands (delta, theta, alpha, and sigma band), younger subjects showed CVE distributions significantly biased to high values compared to older subjects. Remarkably, these distributions were more extreme for delta and sigma bands, respectively, matching frequency bands of the main features of NREM sleep: Delta waves and sleep spindles (Figure 6).

## Discussion

### Conventional analysis of sleep architecture

The present study revealed a lower delta power and distinct sleep architecture in older women compared with younger women; reduced SWS, TST, and sleep efficiency, with increased N1 and wakefulness. These results are consistent with reported features of sleep in older subjects, both men and women (Landolt et al., 1996; Li et al., 2018; Ye et al., 2020). In accordance with previous studies (Brezinová, 1975; Schlemmer et al., 2015), the numbers of SWS episodes were comparable between the two age groups, whereas the SWS duration was shortened in older subjects. Stage transition analysis revealed a decreased ability to maintain SWS, and an increased probability of remaining in wake state; i.e., transitions from wake to wake. Sleep fragmentation is a common symptom of poor sleep quality observed in older women (Lu et al., 2000; Lim et al., 2014). The occurrence of K-complexes during N2 was significantly decreased in older subjects compared with young subjects, consistent with findings from previous studies (Wauquier, 1993; Crowley et al., 2002a,b). Delta wave and K-complexes are thought to be generated by the same mechanisms (Crowley et al., 2002b), and the effects of age on K-complex production can be interpreted as reflecting an age-related change in thalamocortical regulatory mechanisms to induce delta waves (Crowley et al., 2002b). K-complexes may be useful biologic markers of the changes in the nervous system that occur with aging (Crowley et al., 2002b).

### Sleep stage transition

In a fine-grained epoch-by-epoch analysis of sleep stability, the present study revealed differences in the sleep stage transitions between older and young individuals. The significantly more frequent transitions from wake to N1, N1 to wake, N1 to N2, N2 to N1 in older subjects are consistent with findings from a previous study (Schlemmer et al., 2015) in which sleep episode transitions were analyzed at lower resolution. Stage transition analysis in the present study further revealed a lower number of transitions to maintain SWS and a higher probability of remaining awake in older subjects compared with young subjects, i.e., older subjects stayed in SWS for a shorter duration, and in a wake stage for a longer duration of time. These findings indicate a general decrease in stability of sleep architecture and corroborate the loss of SWS in older individuals.

### Delta power and envelope analysis

Interestingly at high temporal resolution, EEG patterns were significantly more homogeneous and less varied in older compared to young subjects. This reduced variability was accompanied by a reduction in EEG delta power in older participants. The amplitude of delta waves depends on the NREM stage. In contrast to the clearly separated amplitudes between N2 and SWS in young subjects, we found an overlap in older subjects, likely underlying the unstable SWS. To assess oscillatory stability, we used CVE analysis to determine the instantaneous power in different EEG spectral bands. Envelope analysis, in particular CVE analysis, is gaining a relevant role among various analysis tools for its specific ability to account for morphological aspects of the signal that are outside the reach of traditional techniques based on Fourier analysis (Díaz et al., 2018; Segning et al., 2022). From early theoretical development in the analysis of local field potentials in animal models (Díaz et al., 2007) to recent applications in the analysis of the human alpha rhythm (Hidalgo et al., 2022), this methodology has shown diverse applications including its use as a clinical diagnostic tool (Díaz et al., 2014; Qian et al., 2021). Regarding the particular goals of this study, we have previously used CVE with the same objective, namely, as a scale-independent measure of the stability of delta waves, with high CVE values indicating unstable oscillations (Park et al., 2021). In general, clinical EEG studies can benefit from CVE analysis, as it provides an alternative approach to classical spectral analysis, much-needed for information extraction from complex EEG morphology not strictly sinusoidal as the basis of Fourier analysis (Cole and Voytek, 2017).

Decreased sleep state stability in older subjects is thus paired with delta oscillations that are less varied and more Gaussian in their temporal energy distribution. This was particularly visible in the lower CVE of delta waves during N2 in older subjects. The difference in CVE in N2 between the age groups may be related to different frequencies of K-complexes, which increase the CVE as abrupt huge slow oscillations in EEG (Li et al., 2018). In the present study, CVE values were positively correlated with K-complexes (*r* = 0.89; *p* < 0.001). Although there is some controversy regarding the role of K-complexes to trigger transitions from N2 to SWS, previous studies provided strong evidence that K-complexes induce neuronal silence (“down-states”) and are the forerunner of delta sleep (De Gennaro et al., 2000; Cash et al., 2009). We expanded the CVE analysis to other EEG bands that are relevant for sleep. We found reduced CVE values in older subjects in all of these bands, indicating that this is a general feature and not restricted to the delta band. This more monotonous EEG of elderly participants indicates that some of the richness of EEG features that can be found in young subjects is lost in aged subjects. The underlying causes for this reduced EEG repertoire obviously need further investigation. As it stands CVE analysis can provide an easily visualized measure of oscillatory stability and the reduced values observed here might provide a general measure of brain aging.

### Potential mechanisms of age-related changes in sleep

Age-related changes in sleep may be related to age-related changes in lifestyle or age-related changes in physiology. Physical activity during the daytime is significantly reduced in older subjects compared with that in young subjects (Husu et al., 2021). The physiologic mechanisms underlying fragmented and suppressed SWS in older subjects are elusive. The number of galanin neurons in the ventrolateral preoptic nucleus, which play a critical role in the regulation of NREM sleep, is reduced in older subjects (Lim et al., 2014). The number of galanin neurons at the time of death is negatively correlated with sleep fragmentation monitored before death. The relation between galanin neurons, and the delta power and stability of SWS remains to be clarified. Another possibility is age-related changes in the sensitivity to corticotropin-releasing hormone, which stimulates the secretion of adrenocorticotropic hormone and cortisol, and suppresses SWS (Vgontzas et al., 2001). Pyramidal neurons in the cerebral cortex fire synchronously in deep sleep or SWS, and the intensity of their synchronous firing correlates with sleep depth (Neske, 2016). Pyramidal cell density decreases with age in all four hippocampal sectors (CA1 through C4), with maximal loss in sector C4. The age-related loss of pyramidal cells is most overt after age 65 (Mani et al., 1986).

### Limitation of the study

In this study, we examined NREM sleep of older (≥62 years of age) women without sleep disorders compared with younger women in their 20 s. To generalize our findings on SWS sleep changes as a characteristic of older people, it will be critical to conduct the same experiment targeting females with sleep disorders as well as males with or without sleep disorders. In addition, it is important to examine sleep in middle-aged subjects to determine the age at which the changes occur.

## Conclusion

In older subjects, although the number of transitions into SWS was almost the same as that in young subjects, the SWS stage was less stable and the duration of SWS over the entire night was shorter. The CVE of delta waves, an index of delta wave stability, was similar between age groups. In older subjects, the delta power amplitude during SWS was lower, and its distribution overlapped with that of N2, which underlies less stable SWS.

## Data availability statement

The raw data supporting the conclusions of this article will be made available by the authors, without undue reservation.

## Ethics statement

The study protocols were approved by the Ethics Committee of the University of Tsukuba (Ref Nos., Tai 29-29 and 30-134), and conducted in accordance with the Helsinki Declaration. The patients/participants provided their written informed consent to participate in this study.

## Author contributions

IP, JS, TO, and KT designed the experiment. CK, JS, SZ, AU, and HO performed polysomnographic recording of sleep. IP, RM, KH, CS, YS, JD, and KV performed the sleep analysis. JS performed the statistical analysis. IP, CK, AI, JD, KV, and KT interpreted the results and wrote the manuscript. All authors contributed to the article and approved the submitted version.

## Footnotes

1
http://r-forge.r-project.org/projects/signal/

2
https://www.stat.go.jp/data/nihon/back15/21.html
